# A Mindfulness-Based Stress Reduction Program via Group Video Conferencing for Adults With Cerebral Palsy – A Pilot Study

**DOI:** 10.3389/fneur.2020.00195

**Published:** 2020-04-03

**Authors:** Helene Høye, Reidun Birgitta Jahnsen, Marianne Løvstad, Jeanette Folkvord Hartveit, Hilde Sørli, Sveinung Tornås, Grethe Månum

**Affiliations:** ^1^Department of Research, Sunnaas Rehabilitation Hospital, Nesodden, Norway; ^2^Department of Clinical Neurosciences for Children and Research, Oslo University Hospital, Oslo, Norway; ^3^Center of Habilitation and Rehabilitation Models and Services (CHARM), University of Oslo, Oslo, Norway; ^4^Department of Psychology, Faculty of Social Sciences, University of Oslo, Oslo, Norway; ^5^Faculty of Medicine, University of Oslo, Oslo, Norway

**Keywords:** disability, pain, stress, coping, mindfulness, e-health, cerebral palsy, adult

## Abstract

**Purpose:** Adults with cerebral palsy experience challenges related to lifelong disability, such as stress, fatigue, pain and emotional issues. E-health services can be delivered regardless of residence and level of functioning. The aim of this pilot study was to explore the potential benefits and feasibility of a mindfulness-based program delivered to adults with cerebral palsy via group video conferencing.

**Methods:** Six adults with cerebral palsy received an 8 week mindfulness group-based program via video conferencing. A multiple single-case study design was applied, including quantitative and qualitative elements. Pain was assessed 16 times through the study period. Questionnaires were administered to gather data on pain catastrophizing, stress, fatigue, emotional distress, positive and negative affect, and quality of life. A focus group interview addressed experiences with the intervention and the mode of delivery.

**Results:** The participants' pain levels showed varied trajectories. Pain catastrophizing and negative affect were statistically significant decreased. Qualitative data indicated benefits from mindfulness in coping and stress management. The video conferencing delivery was evaluated as feasible, with no major adverse effects.

**Conclusion:** Since the pilot study had a small sample size, potential treatment benefits should be interpreted with caution. However, this pilot study provides important information in the planning of future larger and controlled studies on mindfulness-based interventions programs via video conferencing for adults with cerebral palsy and other persons living with long-term disability.

## Introduction

Cerebral palsy (CP) is an umbrella term covering a group of motor impairments resulting from an early brain lesion (1). CP is often accompanied by disturbances in sensation, cognition, perception and behavior, and secondary problems in the musculoskeletal system (1). The prevalence of CP is approximately two per 1,000 births (2, 3). Many people with CP experience secondary problems with increasing age, such as chronic pain, fatigue, and deterioration of function (4–8). As many as 28–67% of adults report chronic pain to the degree that significantly affects mastery and participation, which poses long-term stress factors to the individual (6, 7).

Follow-up programs for individuals with CP have typically had a predominant focus on development and preservation of motor skills, while somatic symptoms and psychosocial factors have been largely ignored (9). Based on a narrative review on adults with CP of factors related to mastery of their disability and health with age, their main concerns were need of social support, self-acceptance and acceptance by others, adaptations in everyday life, and health-care services related to the disability (10). By this, several studies have highlighted the need for complementary intervention programs that enhance self-regulation of physical and emotional well-being (11), counteract loneliness (12), and facilitate the coping potential of the individual (4, 9, 10). This also applies to programs that target long-term pain among persons with CP, where standard medically oriented interventions are typical (6, 7, 13). A study addressing CP-related pain, however, found that having catastrophizing thoughts about the pain negatively affected daily functioning and was associated with depression (4). Therefore, there is a need for more research on coping strategies and psychologically oriented interventions in this patient group.

The recognition of the individual's coping potential is central to the biopsychosocial model, which promotes that biological, psychological, and social factors are interactively involved in health and well-being (4, 14). Coping strategies are described as the repertoire of responses the individual has to manage thoughts, feelings, and actions in demanding and stressful situations (15, 16) The coping strategies are influenced by how the person understands and interprets the situation, perceived source of stress, locus of control, sense of self-efficacy, and by access to social support. According to Sahler and Carr (16), coping strategies can be taught explicitly or through modeling, and therefore have a potential for lifelong development. Facilitating adaptive coping for fatigue, pain, and stress associated with living with CP might therefore be an important part of holistic rehabilitation approaches to this group.

Mindfulness-based stress reduction (MBSR) is a standardized program, that aims at enhancing aspects of coping with distress (17, 18) and disability in everyday life (19, 20). MBSR trains the capacity for conscious presence in the here and now of mind and body, and to adopt a non-judgmental and accepting attitude toward emotions, thoughts, and bodily sensations. In this perspective, mindfulness can be seen to aid individuals in making a more realistic evaluation of stressors. Also, when in a mindful state, it gives the individual a potential to act more purposefully and flexibly to stressors (21, 22). Studies of mindfulness-based interventions in patient groups such as cancer, fibromyalgia, irritable bowel syndrome, and osteoarthritis patients, have shown that the approach can positively influence symptoms, including pain (23–26), fatigue (27, 28), depression, negative affect, and anxiety (29, 30). A study of a mindfulness-based intervention (MiYoga) for children with CP found significant positive effects on better-sustained attention and fewer impulsive errors, but no effects on psychological well-being, quality of life, or physical function (31). Thus, because MBSR has a wide range of positive health benefits in various patient populations, we wanted to explore the usefulness of MBSR for adults with CP.

The existing literature on VC-based interventions indicates that, when effective, the technology can contribute to improved service accessibility (32) regardless of living area and physical mobility. VC might also reduce the patients' use of time, energy, and costs (33). Thus, providing in-home interventions might be particularly beneficial for adults with CP, by increasing accessibility despite limitations in mobility. We are not aware of prior studies describing either an MBSR intervention alone or MBSR delivered by VC in adults with CP.

The main aim of this pilot study was to explore the benefits of a group- and VC-based MBSR program in adults with CP, with respect to the self-reported experience of pain, emotional distress, quality of life, and coping. A secondary aim was to evaluate the feasibility of this particular mode of service delivery, including the participants' experiences with the technical solution applied.

## Materials and Methods

### Design

This pilot study applied a descriptive multiple single case design and included both quantitative and qualitative data.

### Participants

The study was advertised at the Sunnaas Rehabilitation Hospital (SRH) in-patient program for adults with CP, SRH's website, and at the Norwegian CP association's website and paper magazine. Potential participants were referred from their general physicians, and assessed for eligibility by the physician and psychologist who led the MBSR intervention (authors GM and HH). Inclusion criteria were: minimum 18 years of age; with uni- or bilateral spastic CP (34) and gross motor function level (GMFCS) (35) I–IV; pain of at least 3 months with an average score last week of minimum 3 on the numerical rating scale (NRS) (36); movement of the arms and neck to a degree that allowed performance of yoga exercises; and sensory-motor and communicative skills that enabled group-participation via VC. Exclusion criteria were intellectual disability, severe ongoing mental illness and drug abuse. All participants provided written, informed consent to participate before the data collection. The study was approved by the Patient Safety Officer at SRH and by the Regional Committee for Medical Research Ethics, South-Eastern Norway (216/962). The participants have been anonymized and given pseudonyms.

### Data Collection Procedures

**Figure 1:**
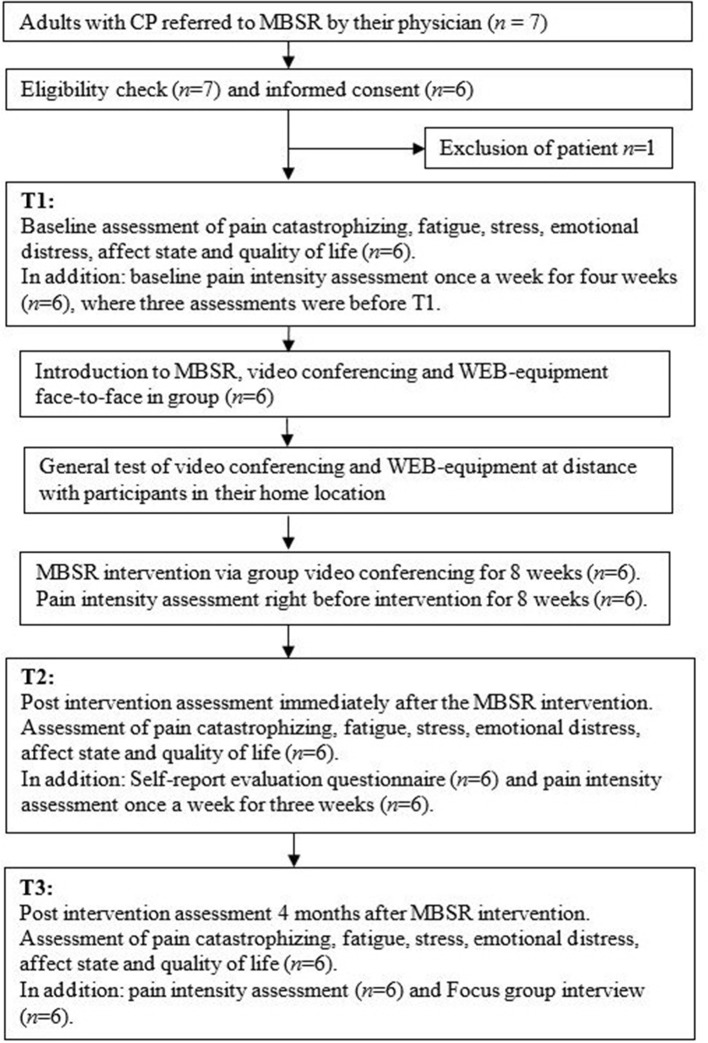
Flow-chart for logistics during the study period.

The project was conducted at or from SRH with the participants physically attending SRH at T1 and T3 (Figure 1). Baseline assessments with self-reported questionnaires was performed before (T1), immediately after (T2), and 4 months after the intervention (T3). Current pain intensity was assessed weekly on the same day (*n* = 1–16) between 12 and 2 PM throughout the study period (Figure 1). A custom made evaluation questionnaire regarding the intervention and experience with use of VC was administered at T2. An audio-recorded focus group interview was conducted at T3 and carried out by the interventionists (authors HH and JFH), and the person responsible for technical equipment and support (author HS).

### Baseline and Outcome Measures

Sample characteristics, and intervention effects were explored with the following measures:

*Pain intensity* was assessed on a 0–10 Numeric rating scale (NRS), where 0 is no pain, and 10 is unbearable pain. According to Breivik et al. (37), mild pain is classified as a score of 1–3, moderate pain from 4 to 6, and severe pain from 7 to 10. A score of ≥3 was used as a clinical cut off (37, 38).

*The Pain Catastrophizing Scale (PCS)* (39) assesses catastrophic thinking about pain. It consists of 13 items and is translated and validated in Norwegian. Internal consistency assessed by Cronbach's alpha were 0.9 (40). Answers are provided on a five-point scale (0–4), with a maximum score of 52, with ≥30 considered as clinically relevant cut-off.

*The Perceived Stress Scale (PSS 14)* (41, 42) is a 14-item questionnaire developed to measure the degree to which situations in one's life are appraised as psychologically stressful. Cronbach's alpha of 0.76–0.78 has been reported (41). The maximum total score on the 14 items is 56. A score of ≥25 was used as clinical cut-off (43).

*The Fatigue Questionnaire (FQ)* (44, 45) is an 11-item questionnaire that assesses physical and mental fatigue, and also provides a total score. Internal consistencies assessed by Cronbach's alpha were 0.73 (MF), 0.86 (PF), and 0.86 (TF), respectively (45). Responses are scored on a scale from 0 to 4, and a total score of ≥16.8 (1.0 SD above normative the mean) was used as a clinical cut-off (46).

*The Hospitality Anxiety and Depression Scale (HADS)* (47) assesses symptoms of anxiety (HADS-A) and depression (HADS-D) in medical and psychiatric settings and the general population, and has been validated in Norway. A review showed average Cronbach's alpha of 0.82 (anxiety) and 0.83 (depression) (48). Both HADS-A and -D consist of seven questions which are scored on a scale from 0 to 3, giving a maximum score for each subscale of 21. A cut-off of ≥8 was considered as the clinical relevant cut-off for both anxiety and/or depression (48).

*The Positive Affect and Negative Affect Schedule (PANAS)* (49) was used to assess positive (PA) and negative (NA) affect. We used the state version, where participants are asked to indicate to what extent they experienced each of the named adjectives at the time of assessment. PANAS has good psychometric properties, with Cronbach's alpha of 0.85 (PA) and 0.90 (NA) (50). Norms from a general UK population were used [mean score for NA = 16 (SD 5.5) and mean score for PA = 31.5 8 (SD 7.65)] (46). On the basis of the UK population study, NA ≥ 21.5 (1 SD above the normative mean in the UK study), and PA ≤ 23.85 (1 SD below the normative mean in the UK study) were used as clinical cut-off (46).

*The Perceived Quality of Life Scale (PQoL)* (51) was used to assess the quality of life. The PQoL questionnaire consists of 19 items, where the mean score is reported, and 10 is the highest score. In a Norwegian validation study (52), the Cronbach's alpha was 0.93. From that study, the mean score in a healthy population was 7.1 (SD 1.2). A score of ≤5.9 (1.0 SD below the normative mean in the same study) was considered as clinically relevant cut-off.

The evaluation questionnaire consisted of 12 custom made questions with fixed response alternatives and spaces for open comments, asking about satisfaction with the intervention, the benefit of it in everyday life, and their experience with VC (Table 4). The responses were subsequently used to develop an interview guide to be used in the focus group interview. A focus group interview approach was selected because the method is suitable for incorporating a reflexive process about themes, to explore the breadth and exchange of opinions and experiences in a group, and identifying what might represent shared experiences among the participants (53, 54).

### Mindfulness-Based Intervention

The introduction to MBSR was given to the group face-to-face at SRH and included training in the use of the technical equipment. After that, the participants received weekly VC-based MBSR in their homes over 8 weeks. Table 1 describes the different themes and sessions. Participants were connected to a closed web-group on their PC, with web-camera, speakerphone, and VC software installed. They could see and hear the instructors and the other participants, ask questions, and make comments. The online sessions were conducted from SHR by the MBSR certified psychologist (author HH) and yoga certified physiotherapist (author JFH), Both authors were highly experienced with using MBSR and yoga to different patient groups in the field of neurological rehabilitation at SRH.

**Table 1:** MBSR session overview.

- Welcome by instructors
- Participant exchange of experience (related to mindfulness and the theme from previous session)
- Formal exercise (breathing anchor, body scan, visualization exercises or yoga)
- Theme:
• Week 1: Mindfulness: what it is and how you do it
• Week 2: Attention and awareness: to be present
• Week 3: Stress: responding vs. reacting
• Week 4: Pain: responding vs. reacting
• Week 5: Feelings and worries: how to deal with it in a mindful way
• Week 6: Mindfulness in everyday life
• Week 7: Self-Compassion and loving kindness
• Week 8: Summary and how to develop your own further practice
- Questions from participants
- Formal exercise (breathing anchor, body scan, or yoga)
- Homework (what to focus on the coming week; formal and informal exercises)

*In each session, there was a brief introduction to a theme central to mindfulness, participant exchange of experience, formal exercises, and introduction to exercises, such as eating or moving mindfully*.

The MBSR program was accommodated and modified to the technological solution and the CP population in the following ways: (a) each session was shortened from 2 to 1.5 h; (b) there were no full-day retreat or instruction on writing diaries; (c) yoga exercises were performed from a sitting position; (d) information was provided on stress and pain physiology and the importance of mental factors in modulation of pain signals; and (e) topics related to fatigue and concentration were included. The participants received written summaries of each session; they were encouraged to practice formal exercises daily and received audio files with instructions of the body scan and the breathing anchor exercises.

### Technology and Safety

The Unit for Technology and eHealth at SRH was responsible for the technical solutions and support. The Acano/Cisco Meeting App (www.acano.com) was used via a technological platform offered by the Norwegian Health Network. This end-to-end encrypted software met the Norwegian government's requirements for secure telecommunications. All participants provided written confidentiality agreements and consented to sit alone under the MBSR-sessions, except for one, who also provided written consent allowing a personal assistant to be present. The assistant provided a confidentiality agreement. The online personnel at SRH had phone numbers to relatives and emergency services. The psychologist leading the intervention could be contacted between sessions if needed and she consulted the study physician (author GM) if necessary. Adverse events, including technical problems, were logged.

### Analysis

Descriptive statistics were generated for the sample characteristics and outcome variables. One pain assessment was missed once for one participant during the intervention and was estimated based on the average pain of the two closest assessments (before and after). Although the small sample size limits the power of the statistical analysis, we used the Wilcoxon Signed Rank Test to explore changes between baseline T1, T2, and T3, with *p* < 0.05 being considered statistically significant. The statistical analyses were conducted using SPSS v.22 (IBM Corporation, Armonk, NY, USA).

Qualitative data were analyzed using thematic analysis, to capture important aspects of the data in relation to the research question, and identify levels of patterned response or meaning within the data set (55, 56). The first steps consisted of listening to the audio-recording and getting an overall sense of what was communicated verbally and emotionally. A detailed verbatim transcript was checked against the recording to ensure accuracy, together with the written comments from the evaluation questionnaire of the intervention. The focus group interview consisted of two data sets. In the first data set (experience of outcome of intervention), we generated initial codes for the semantic meaning in the text, secondly reviewed and refined themes in the form of text extracts of each theme, and then defined and named the themes. In the second data set (experience and evaluation of the use of VC) we based the thematic analysis on the themes in Banbury's research (32), highlighting aspects of feasibility and acceptability, such as usability, communication adaption, and accessibility. Since the data from the focus group interviews were a deepening of the written comments in the evaluation questionnaire, these data are reported together.

## Results

### Participants

Five persons meeting all inclusion criteria and one person without an active pain problem, but with distinct fatigue and emotional distress, consented to participate in the study and were included. One person was excluded due to psychiatric issues. Table 2 summarizes the demographic characteristics of the six participants. All participants, two men and four women, were Caucasian, and the median age was 34 years (range 20–50). Four persons had independent walking ability in most settings, and two were wheelchair users. All had more than 12 years of education, and all except one were in part-time (*n* = 3) or full-time (*n* = 2) paid jobs. Four persons were single, and two of them were living alone. Two persons were on their usual antispastic- (Lioresal) or analgetic- (paracetamol) peroral medication throughout the study period, tree persons used no medication and one person took up her previous antispastic medication due to increased spasms.

**Table 2:** Participant characteristics.

**Participant**	**CP diagnosis**	**GMFCS level**	**Marital status**	**Age**
1. Theo	Spastic unilateral	I	M, C	30-ies
2. Anna	Spastic bilateral	II	S	30-ies
3. Mimmi	Spastic bilateral	IV	M, C	40-ies
4. Dora	Spastic bilateral	IV	S	40-ies
5. Wilmar	Spastic bilateral	II	S	20-ies
6. Ronja	Spastic unilateral	II	S	20-ies

*GMFCS, gross motor function classification system; Marital status, M = married or cohabitant, C = children and S = single*.

### Symptom Assessments

**Figure 2:**
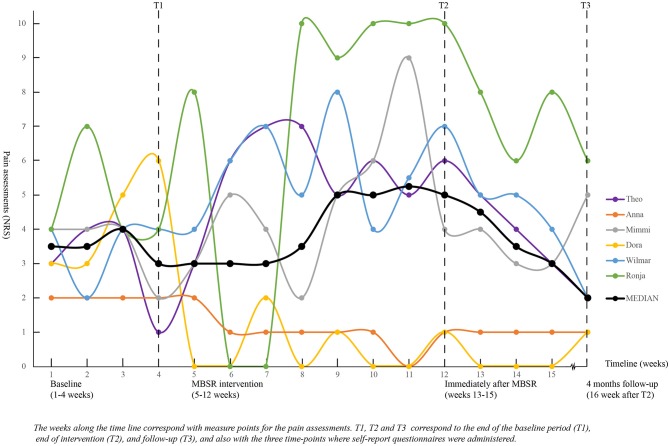
Pain assessment during the study period for all six participants.

The median pain intensity at baseline was 3.3 (range: 2.0–4.3). No statistically significant differences were found between pain intensity at baseline and at T2 or T3 (data not shown). The multiple assessments of pain (*n* = 16) demonstrate each participant's trajectory over the study period. As shown in Figure 2, pain varied both within and between participants.

Table 3 shows the assessments of pain catastrophizing, stress, emotional distress and quality of life.

**Table 3:** Individual and group results on self-report questionnaires at T1, T2, and T3.

	**Time**	**Theo**	**Anna**	**Mimmi**	**Dora**	**Wilmar**	**Ronja**	**Median value**	**Inter-quartile Q1, Q3**	**Range**
Pain catastrophizing	T1	13	4	21	12	26	36	17	10.0, 28.5	4–36
	T2	6	5	24	9	22	45	15.5	5.8, 29.3	5–45
	T3	11	3	20	9	17	28	14*	7.5, 22.0	3–28
Stress	T1	26	33	33	36	34	44	33.5	31.0, 38.0	26–44
	T2	28	29	34	27	29	34	29	27.8, 34.0	27–34
	T3	34	25	34	34	33	38	33.8	31.0, 35.0	25–38
Fatigue	T1	14	24	21	11	17	13	15.5	12.5, 21.8	11–24
	T2	11	19	23	5	16	28	17.5	9.5, 24.3	5–28
	T3	11	13	21	5	20	14	13.5	9.5, 20.3	5–21
Anxiety	T1	2	15	9	9	10	11	9.5	7.8, 12.0	2–15
	T2	2	7	10	6	12	14	8.5	5.0, 12.5	2–14
	T3	4	6	9	0	13	13	7.5	3.0, 13.0	0–13
Depression	T1	0	7	4	5	7	4	4.5	3.0, 7.0	0–7
	T2	0	2	4	4	10	1	3	0.8, 5.5	0–10
	T3	1	1	3	4	3	1	2	1.0, 3.3	1–4
Negative affect	T1	12	26	13	11	15	45	14	14.0, 30.8	11–45
	T2	10	14	22	12	16	39	15	11.5, 26.3	10–39
	T3	10	10	10	10	13	41	10*	10.0, 20.0	10–41
Positive affect	T1	32	22	41	46	19	35	33.5	21.3, 42.3	2–46
	T2	33	32	39	50	21	28	32.5	26.3, 41.8	21–50
	T3	33	37	35	50	26	33	34	31.3, 40.3	26–50
Quality of life	T1	6.4	5.8	5.4	5.8	4.7	7.5	6.4	5.2, 6.6	7.5
	T2	6.1	7.4	4.2	6.8	4.9	2.5	6.1	3.7, 7.0	2.5–7.4
	T3	6.4	6.7	5.4	8.2	5.6	6.1	6.4	5.3, 7.0	5.4–8.2

*Results are expressed as raw scores. Pain catastrophizing, Pain Catastrophizing Scale (PCS); Stress, The Perceived Stress Scale (PSS-14); Fatigue, The Fatigue Questionnaire (FQ total); Anxiety, Hospitality Anxiety and Depression Scale (HADS-A); Depression, Hospitality Anxiety and Depression Scale (HADS-D); Negative affect, Positive Affect and Negative Affect Schedule (PANAS state negative); Positive affect, Positive Affect and Negative Affect Schedule (PANAS state positive); Quality of life, The Perceived Quality of Life Scale (PQoL)*.

* *Difference T1–T3 sign. at p < 0.05*.

During the study period (from T1 to T3) there was a statistically significant decrease in pain catastrophizing, and in negative affect (*p* = 0.03).

### Evaluation Questionnaire

All participants except one experienced subjective benefit from the MBSR program, and would recommend it to other persons with CP (see Table 4, questions 1 and 5). The participant evaluating no/little benefit elaborated in the focus group that the program did not meet his expectations regarding pain relief, which was the reason why he would not recommend it. All six participants were satisfied with the form and content of the intervention, as well as receiving it via VC. The majority wanted more sessions, and one person wanted a video of the yoga exercises to assist the homework.

**Table 4:** Custom-made evaluation questionnaire.

1	What benefit did you have from the MBSR sessions?		Some/great: 2, 3, 4, 5, 6*	No/little: 1
2	Was the theory/purpose of the MBSR clearly explained to you?		No:	Yes: 1, 2, 3, 4, 5, 6
3	Were the exercises clearly explained to you?		Yes: 1, 2, 3, 4, 5	No:
4	Did you get enough practice in formal techniques in the sessions?		Yes: 1, 2, 3, 4, 5, 6	No:
5	Would you recommend MBSR to other people with cerebral palsy?		Yes: 2, 3, 4, 5, 6	No: 1
6	Did you miss more or better defined home tasks?		Yes: 1	No: 1, 2, 3, 4, 5, 6
7	Did you use the MBSR-exercises outside the intervention sessions?		Yes: 1, 2, 3, 4, 5, 6	No:
8	How was the progress in teaching?	Too slow:	Satisfactory: 1, 2, 3, 4, 5, 6	Too fast:
9	How was the amount of MBSR sessions?	Too few: 2, 3, 4, 5	Satisfactory: 1, 6	Too many:
10	How intense were the MBSR sessions?	Too low:	Satisfactory: 1, 2, 3, 4, 5, 6	Too intense:
11	How was it to get the MBSR via web?		Satisfactory: 1, 2, 3, 4, 5, 6	Not satisfactory:
12	Was there something you missed during the MBRS sessions?		Yes:	No: 1, 2, 3, 4, 5, 6

* *The numbers represent individual participants that provided this specific response; 1 = Theo, 2 = Anna, 3 = Mimmi, 4 = Dora, 5 = Wilmar, and 6 = Ronja*.

### Evaluation Questionnaire and the Focus Group Interview -Experience of Outcome (Benefit) of the MBSR

In line with the questionnaire data presented above, all participants evaluated the intervention as useful regarding multiple aspects of everyday coping and stress management. The themes that emerged were all associated with a superordinate theme of *coping benefits*. Moreover, subthemes that emerged were (I) *knowledge of CP and secondary symptoms*, (II) *acceptance of bodily limitations and resources*, (III) *regulation of emotions*, (IV) *regulation of activity, and rest* and (V) *communication of needs, limits and resources*. These themes had a reciprocal relationship with each other and the superordinate theme.

#### Knowledge of CP and Secondary Symptoms

The knowledge included learning more about CP and common challenges related to spasms, pain, fatigue, and cognition, from each other and the instructors, particularly for those who had not received CP-oriented health care provision for many years:

“*People immediately think that I must know everything about CP. I don't! It's the way I was born, the way I've always been. It's my normal situation.”* [Anna]

One participant experienced what she first expressed as “bodily shakings” with accompanying pain during the MBSR-intervention. She learnt from the study physician that she was experiencing spasms, and realized that she had ignored these symptoms.

“*I didn't know what was going on with me. Is it an anxiety attack? Now I know I have spasms because there is something physically wrong with me. I have learned so much, even though it has been a painful process.”* [Ronja]

#### Acceptance of Bodily Limitations and Resources

Increased body awareness and acceptance were commented upon from several as an important benefit, typically expressed as “*better at taking seriously the signals my body sends me”* and “*acceptance of the situation.”* This included being aware of sensations in the body, such as pain, which might reflect for example an imbalanced activity level.

“*It was on account of the pain that I signed up for the course, and it has not helped with that. But I have achieved other things. … What the pain tells me is that there is something wrong with my level of activity. Now I know I ought to work two days instead of three.”* [Leo]

One of the two persons who reported increased pain during MBSR said that it was a new experience to consciously attend to bodily signals, which resulted in temporarily increased pain. This participant received three follow ups with one of the interventionists.

“*It was frightening when the pain increased… But I believe I have been shutting out a whole lot. I may have the pain, but don't focus on it. … So I have to take tiny little steps in teaching myself mindfulness.”* [Mimmi]

Several talked about increased awareness and acceptance that their overall capacity and (psychomotor) speed may be decreased, typically expressed as “*everything goes a little more slowly*.” Also, all participants experienced increased awareness of their use of compensatory strategies in everyday life.

“*Because we have CP, we use different strategies than others to get where we are. I have not been so aware of this before … It feels like we must do more all the time for fear of it not being adequate.”* [Wilmar]

#### Regulation of Emotions

All participants gave examples related to changes in emotional regulation, which included stopping negative self-thoughts, rumination, and staying calmer in stressful situations.

“*I manage to let trivial things be trivial things and challenges be challenges. I manage to stay calm when things get tough, that is to say, in difficult situations in which I used to lose my temper and vent my anger and frustration.”* [Dora]

To stop and focus on breathing was helpful in emotional regulation for several.

“*I begin crying almost uncontrollably if I feel stressed, am sorry about something, or am extremely happy. … I had an episode where I got angry at a person for a good reason and began to cry. Then I said: ‘Just breathe, just breathe!' And I took some breaths until I felt fairly steady. Then I yelled at him: ‘Give me a minute to finish crying and calm down, and then I'll deal with it.' I haven't done that before.”* [Anna]

Less rumination and a feeling of being relaxed when going to bed were also reported.

“*In the past, when I went to bed, my head was filled with thoughts, and I lay there brooding. I don't have that problem any longer. Now I have taught myself to turn off the switch.”* [Dora]

#### Regulation of Activity and Rest

All participants reported that the intervention had made them more aware of their need to take breaks when necessary, with a statement such as “*now I take those five minutes,”* which made them feel more relaxed, refreshed or in control.

“*I sneak in a few extra breaks on the job. If I use the toilet, I stand and look at myself in the mirror and take some deep breaths before I go out again, so that I feel like it has been a real break.”* [Anna]

Some told about becoming more conscious to not spend all their energy at once, getting more focused on prioritizing and coping with aspects of attention and peace, as well as cutting back on their need to be perfect.

“*This course has helped me to be able to focus, to be focused only on the task at hand, complete it, and not think I have to do everything at super speed. … I have also become more conscious of thinking about what I shall prioritize.”* [Ronja]

#### Communication of Needs, Limits, and Resources

The analyses revealed increased awareness and ability to talk about their limits, boundaries, abilities and needs. This was mainly to employers and friends, but also health personnel, because of what was reported as an increased understanding of their health needs due to CP. One individual was requested to perform a task at work and said:

“*My old self would have said ‘yes' and thought that if I didn't say ‘yes', I would lose my job. Instead, I replied: ‘I'm still a little behind because I've been helping some of my colleagues. Is it all right if I start this new task a week later?' It worked out fine.”* [Anna]

Another individual who was in the process of reducing her working hours due to CP-related symptoms had agreed with her boss to speak out more clearly about her true capacity for work.

“*I have promised him* [the boss] *that we'll have honest communication. I'll not say I'm doing fine if things are not fine. So in a way it has been easier to make accommodations* [on the job] *after this course.”* [Ronja]

### Feasibility and Experience With Group- and VC-Based MBSR

In the qualitative evaluation of the technical aspects of VC-delivered treatment, the main themes were feasibility and acceptability of technical equipment and solution, exposure and security, communication and social connectedness, and accessibility and adherence.

#### IT Usability, Training, and Support

There was consensus that the initial technical training at SRH was necessary to overcome worries about technical mastering and barriers.

“*I don't believe it would have been possible to start this project without having gone through that [the technical aspects] beforehand. … One could feel confident …”* [Ronja]

During the VC sessions two participants reported temporary problems in switching the web-camera on, to adjust the microphone, and some episodes of freezing picture and software problems. Technical problems were not of a magnitude that caused interruption of the sessions. The flexibility of the ICT support team was appreciated by all of the participants.

#### Privacy and Security

Exposure of privacy in the form of seeing each other's homes was reported as something all participants got used to.

“*I was a little concerned. Oh, no! The laundry is hanging behind me! Sometimes I had to tidy up beforehand … and after a while, it didn't always seem quite so important.”* [Wilmar]

The individual that was troubled by spasms in some VC-sessions felt uneasy about exposing this and agreed with the interventionists to pull a little away from the web-camera when needed. This allowed this person to attend sessions, but also (at least for a period) to hide the need for referral to the physician in the project.

“*It was, of course, a very good arrangement that if the spasms became too bad, I could move away from the camera. But then I realized that it was perhaps not so fortunate after all. … The disadvantage with VC was that I could protect myself. It took longer to get help.”* [Ronja]

No other adverse situations were logged by the interventionists, the ICT team, or the participants. There was a consensus that the IT security in the project was well-taken care of, and participants were pleased with the amount of information provided on this issue.

#### Communication Adaption and Social Connectedness

All participants reported that the initial face-to-face meeting laid an important foundation for communicating more freely on VC. Rules for turn-taking were considered necessary, as well as the fact that the interventionists actively invited participants into the communication. There was a consensus that to communicate via VC was satisfactory. The group size was also considered adequate.

“*I felt that when I was speaking it was like I didn't think there was a screen there. It was a very natural conversation somehow.”* [Anna]

All expressed that they had experienced connectedness in the group. Factors contributing to this where described as “*openness among participants,” “humor,” “trust,” “caring,”* and “*exchange of experience with peers.”*

The closing of the MBSR VC-sessions was experienced as being too abrupt or sudden, resulting in a feeling if anticlimax, where some felt lonely.

“*If we had met face to face … it would have ended naturally. We would have cleared the table, made small talk, chatted a little about this and that. Five minutes would have passed and we would have been ready to move on. But online it is just pushing the off-button. Click! And they were gone.”* [Anna]

#### Accessibility and Adherence

The advantages of CV intervention vs. face-to face at a treatment location, were expressed as “*easier logistics,” “broke down geographic barriers,”* as well as “*saved mental energy,”* and “*less stressful.”* No one reported that they had wanted the intervention face-to-face instead. One participant said adherence on VC was easier on “*bad days.”* VC delivery was essential for participation for half of the participants, due to the reasons expressed above, as well as a lack of geographical availability.

“*Decisive for me was that I never would have had the opportunity to participate without this arrangement. It wasn't possible at my local hospital.”* [Mimmi]

Several stressed the importance of having easy access to individual follow-up during the intervention. The interventionists logged two adverse events due to spasticity and increased pain, which led to individual follow-up between sessions. The majority said that an increase from eight to ten or twelve MBSR sessions would have been appropriate, as learning mindfulness was experienced to be a process that took time to process and to implement into everyday life. The length of sessions was considered satisfactory, except for one participant who wanted shorter sessions due to fatigue. Some reported a need to make it clear before the intervention that it might not lead to pain reduction but improved coping (Table 4, questions 1 and 5).

## Discussion

This pilot study explored the feasibility and results of a group-based MBSR program via VC for adults with CP. The intervention resulted in statistically significant reduced pain catastrophizing and negative affect, while a reduction of pain intensity as such was not obvious. Qualitative data demonstrated benefits in aspects of coping with chronic CP-related symptoms, and that the VC-format provided increased accessibility and social connectedness.

All participants had baseline symptoms of either pain, emotional distress or decreased quality of life, or a combination of these, and half the group had clinically significant fatigue, which is consistent with the known health challenges in adults with CP (10, 57–59). The fact that all participants had a high total stress level highlights that adults with CP experience strain on their coping abilities and adjustments (9, 57).

Catastrophizing thoughts and emotions related to pain are typically understood as the cognitive-emotional experience (39), or reactivity (60) to pain. Reduction of pain catastrophizing has also been found in a Canadian study of a 10 week VC-based MBSR program adapted to chronic pain and delivered to different diagnostic groups (61). The study compared the distant VC MBSR group with a physically present MBSR group and controls. The study indicated that the effect of MBSR on catastrophizing was not hindered by the VC mode of delivery, but that the magnitude of pain reduction might be somewhat lower than when the interventionist is physically present.

In a laboratory setting, Zeidan, and Vago (62) compared experienced and novice meditators with regard to how they rated pain intensity and pain unpleasantness. They found that the intensity of induced pain was rated equally, but that experienced meditators rated the pain as less unpleasant, suggesting that meditation might support non-reactivity to pain. Reduced reactivity to pain might be associated with less catastrophic thinking about pain, which was found in the current study.

An association between pain catastrophizing and negative affect, such as negative attention bias and negative expectation, has been documented (63). One study by Engel et al. (64) found that pain catastrophizing was the coping strategy that most interfered with physical mobility, self-care, recreational, and social activities. Prevention of catastrophizing might thus play a central role in living with chronic pain. This finding in our intervention is in need of replication in larger scale studies.

Some participants experienced increased pain intensity during the intervention. Reasons for this may be multi-faceted. Pain varies naturally, so the results might partly simply reflect natural cycles. However, weekly pain assessments might have contributed to an increased awareness directed toward pain experiences. This might have resulted in an attentional bias, known to have the potential to increase symptoms, especially among pain fearful individuals (65). Also, the intervention itself invites the participants to pay attention to and observe bodily sensations, necessarily giving rise to both comfortable and uncomfortable sensations (66).

The mindfulness literature is sparse about potential symptom increase and unexpected and unwanted effects (UE), such as increased pain and disturbing emotions. However, a multicenter survey of mindfulness and meditation practitioners found that 25% reported UEs; mostly mild and transitory not in need of medical attention. This happened most often in long individual practice in focused attention meditations, and not so frequently in body awareness meditations (67), which dominates MBSR-practice. In a brief MBSR-based program Sass et al. (68) found that high discomfort with emotions, including low tolerance for negative affect, significantly moderated emotional distress reduction. The authors suggest that that the capacity of emotional tolerance should be addressed prior to treatment. This is in accordance with our results, indicating the need to be careful in the selection of VC-based MBSR-candidates. The participants should be informed that the method might not alter pain in itself, and that MBSR may create increased awareness of both comfortable and uncomfortable bodily sensations and feelings before more adaptive coping is developed. However, the qualitative data indicated that even those who experienced more pain felt that the treatment was helpful and that the increased awareness of their functional limitations was for the better in a long-term perspective.

Five interrelated sub-topics of what seemed to be aspects of coping with adult CP appeared from the qualitative data. First, knowledge of CP and secondary symptoms seemed to be a necessary step to decrease insecurity about symptoms, and to enable the use of new coping strategies. Increased knowledge gave participants a platform for acceptance, and communication about their health, resources and needs. This is in line with studies on aging with CP (10, 57), where one review study (10) found that greater knowledge and understanding among individuals with CP improved health-related decision-making. The participants also reported improved skills in communicating their needs and strengths more clearly to employers, friends and health care professionals. In accord with this, Sienko's research (69) on young adults with CP, found that skilled communication of health concerns and needs to medical professionals might enhance locus of control and self-esteem, health, and well-being. According to Mudge et al. (57), to take “charge of help” is central to adults, as the symptoms of CP and secondary conditions will likely change with age, and therefore trigger a need for medical follow-up and adjustments in health care provision.

Also, acceptance of bodily limitations and resources is a significant factor for purposeful coping with CP. One qualitative study (57) showed that acceptance might help the individual to achieve a more realistic picture of what to expect, enabling more positive and adaptive responses to health changes. Brunton and Bartlett (70) describe how bodily awareness, adaptation and regulation among persons with CP is a lifelong process due to changes in symptoms and capacity levels. Increased acceptance seemed to lead to less self-blaming, a connection well-described in positive psychology, where less self-blaming is considered important for better psychological health and more flexible coping (71). Increased acceptance of own strengths and weaknesses and less self-blaming may reflect development of more self-compassion, which was a weekly topic in the adapted MBSR.

When it comes to emotional regulation, the participants described less rumination, being increasingly able to stop negative thoughts and to stay calm in stressful situations. One common factor account for this may be a basic skill in mindfulness training, namely decreased reactivity, where thoughts and feelings are allowed to come and go, without the individual identifying with them or being carried away by them (22, 72, 73). Another strategy for emotional regulation was to establish contact with the “breathing anchor,” which was experienced as effective to stop rumination. This strategy is understood as a form of attentional control, a well-documented aspect of mindfulness that might contribute to downregulation of uncomfortable emotions (74).

The theme “regulation of activity level and rest” also included being aware that bodily symptoms might signal a need to adjust. Improved coping with the need to balance rest and activity, might in turn help reduce strain that is contributing to pain (14). This is an adaptation process that might take more time than the current intervention allowed for. A study of walking children and adolescents with CP and controls, concluded that the strain from walking, close to or above anaerobe threshold for many, might explain fatigue (75). This type of strain is probably present for adults as well. Five of the participants in our study were walking or partly walking, and two of these had fatigue above clinical levels. To take short breaks and include the “breathing anchor” seemed to be helpful to feel bodily and mentally refreshed.

Regulation of attention, in doing one thing at the time, and staying task-focused, was experienced as one of the most important gains from the MBSR-intervention for one participant. This is not surprising, as research on mindfulness and yoga proposes a modification of attentional subsystems in experimental tasks assessing attention in both healthy adults (76) and children with CP (77). This effect might be particularly important in patient groups were attentional capacity is impaired due to brain injury.

### Feasibility and Acceptability of Group- and VC-Based MBSR

Technical training before MBSR intervention and access to support during the intervention was considered necessary. This is in accordance with Banbury et al. (32) who found that this is central to the acceptability of home-based VC in groups. It might also be of particular relevance to participants who, due to CP, have sensory, motor, cognitive, or other challenges that result in a need for individual adaptations. The participants had little concern about privacy issues, such as showing a part of their home along with themselves on the camera, which is also in line with Banbury et al. (32). Surprisingly, the literature is sparse concerning aspects of security in videoconferencing groups, but a Danish article about challenges in future telehealth provision (78) considers this to be a central theme. This pilot study indicates that IT security and the participants' respect for confidentiality are central to establishing a sense of trust, where the group members feel they can communicate freely.

Communication adaptation was evaluated as satisfactory, and only one participant experienced that communicating via VC was cognitively demanding. This is also in line with Banbury et al. (32), who found that only a few felt uncomfortable using VC to communicate with others. Clear guidelines for not interrupting each other and to speak slowly seems to be necessary, and is often a part of VC group protocols. This might be of importance if participants have some motor speech problems, such as in this group, where one participant had slow speech and dysarthria.

The fact that VC did not seem to hinder the establishment of group connectedness is in line with one of the main findings from Banbury et al. (32, 79). High attendance rate, as in our study, might enhance cohesiveness, and also indicates high feasibility. The literature is not conclusive regarding the need to meet face-to-face before VC (32). However, it may take some time before some participants feel at ease with online meetings, and in this study, participants reported that meeting face-to-face was helpful. Also, the interventionists got to know the participants, which made individual adjustments and follow-up between sessions easier. This is in line with Greenhalgh et al. (33), who found that video consultations appear to work better when the clinician and participant already know and trust each other. The VC mode of delivery was appreciated by the participants due to the reduced energy expenditure, time spent on traveling, and because it was easier to combine treatment with work and daily life. This all rendered the intervention accessible to persons who did not have access to this kind of treatment in their communities. These findings are also in line with Banbury et al., who argue that using VC might overcome known mobility, time, and distance related barriers (32). The participant feedback interestingly indicated a need to pay particular attention to how sessions are closed. Closing of VC sessions is absolute, with no help from closing rituals that often accompany face-to-face conversations. Also, some participants might have activated uncomfortable thoughts, feelings and bodily sensations, and need help from an interventionist to downregulate before session closure. The VC format itself might also be a barrier to noticing this type of reactions. To our knowledge, these themes have not been addressed in the literature on group-based VC interventions before.

### Limitations and Strengths

The major limitation of this study is the small sample size and lack of a control group. This hampers the generalizability of findings, and statistical analysis should be interpreted as crude indications of possible areas of interest in future studies. On the other hand, as the literature on the use of VC in the CP-population is limited, it was considered necessary to explore feasibility and efficacy in a pilot study before establishing a large scale randomized controlled study. The group had a high educational level, probably not representative for the overall CP population. The interventionists conducted the focus group interview, which might have affected what the participants chose to share in their evaluations. One strength of the pilot study was that the MBSR-program was led by a psychologist and physiotherapist with longstanding experience with the CP population. This included familiarity with potential challenges such as cognition, emotional issues, and restrictions in movements, which made it easier to individually adapt the VC sessions. In addition, the ICT team consisted of health personnel, and the participants had individual adaptations and training in technical equipment before the intervention. Also, the participants had access to individual follow-up during the intervention, and there were no drop-outs. In addition to qualitative methods, the pilot study used standardized questionnaires for outcome assessment. We are not aware of any other studies exploring MBSR delivered in a group and by VC in adults with CP. Even if the study is exploratory, we consider the results relevant in informing future study protocols.

## Conclusion

This pilot study found that an adapted 8 week MBSR program via VC was beneficial in managing pain catastrophizing and negative affect among adults with CP. The qualitative data indicate that the intervention and VC delivery was feasible with no major adverse effects. Group-based MBSR delivered via VC seems appropriate to reduce energy cost and increase accessibility, and has potential as a supplementary health service program for adults with CP. This pilot study provides important information in the planning of future studies with a more rigorous scientific design on group-based VC intervention programs for adults with CP and other patient groups with long-term disability.

### Recommendations for a Further MBSR Program via VC

Because CP is a complex condition often associated with pain, fatigue, and stress, it seems useful to integrate psychoeducation about these in an adapted MBSR. An increase from eight to ten sessions may, therefore, be fruitful. The closing of each MBSR session on VC needs sufficient time and careful monitoring. It is also important to identify those who may be at risk of experiencing negative treatment effects. This could be achieved through an individual assessment before the intervention, as well as by offering individual follow-up during the intervention. Because qualitative data indicated increased self-acceptance and less self-blaming, which may reflect self-compassion, future studies should include an outcome measure regarding self-compassion.

## Data Availability Statement

The data that support the findings of this study are available from the corresponding author, (HH: helene.hoye@sunnaas.no), upon reasonable request.

## Ethics Statement

This study involving human participants were reviewed and approved by the Regional Committee for Medical Research Ethics, South-Eastern Norway (216/962). The patients/participants provided their written informed consent to participate in this study.

## Author Contributions

HH, RJ, JH, HS, ST, and GM have been part of the research group from the start, and have all contributed to the planning, data collection, and data analysis. First author HH led the intervention, JH was co-therapist in the intervention, while HS was responsible for the technical solutions and support provided. ML has taken actively part in data analysis and writing of the paper. While first author HH has been mainly responsible for writing the manuscript, all authors have contributed significantly in the writing process, and have read the final version of this manuscript.

### Conflict of Interest

The authors declare that the research was conducted in the absence of any commercial or financial relationships that could be construed as a potential conflict of interest.
